# Related factors and use of free preventive health services among adults with intellectual disabilities in Taiwan

**DOI:** 10.1186/1472-6963-14-248

**Published:** 2014-06-12

**Authors:** Suh-May Yen, Pei-Tseng Kung, Li-Ting Chiu, Wen-Chen Tsai

**Affiliations:** 1 Department of Health Services Administration, China Medical University, No. 91 Hsueh-Shih Road, Taichung 40402, Taiwan; 2 Department of Chinese Medicine, Nantou Hospital, Nantou, Taiwan; 3 Department of Healthcare Administration, Asia University, Taichung, Taiwan

**Keywords:** Intellectual disabilities, Disability, Preventive health service, Adult health examination

## Abstract

**Background:**

This study aimed to investigate the utilization of preventive health services in the adults with intellectual disabilities from the nationwide database.

**Methods:**

The research method of this study is secondary data analysis. The data was obtained from three nationwide databases from 2006 to 2008. This study employed descriptive statistics to analyze the use and rate of preventive health services by intellectual disabled adults. Chi-square test was used to determine the relationship between the utilization of preventive health services and these variables. Multivariate logistic regression analysis was used to explore the factors that affect intellectual disabled adults’ use of preventive health services.

**Results:**

Our findings indicated 16.65% of people with intellectual disabilities aged over 40 years used the preventive health services. Females were more frequent users than males (18.27% vs. 15.21%, p <0.001). The utilization rate decreased with increasing severity of intellectual disabilities. The utilization was lowest (13.83%) for those with very severe disability, whereas that was the highest (19.38%) for those with mild severity. The factors significantly influencing utilization of the services included gender, age, and marital status, urbanization of resident areas, monthly payroll, low-income household status, catastrophic illnesses status and relevant chronic diseases and severity of disability.

**Conclusions:**

Although Taiwan’s Health Promotion Administration (HPA) has provided free preventive health services for more than 15 years, people with intellectual disabilities using preventive health care tend to be low. Demographics, economic conditions, health status, relevant chronic diseases, environmental factor, and severity of disability are the main factors influencing the use of preventive healthcare. According to the present findings, it is recommended that the government should increase the reimbursement of the medical staff performing health examinations for the persons with intellectual disabilities. It is also suggested to conduct media publicity and education to the public and the nursing facilities for the utilization of adult preventive health services.

## Background

The global prevalence of intellectual disabilities was 10.37 per 1,000 populations [1]. In the end of 2011, there were 98,046 people with intellectual disabilities, accounting for 0.4% of the total population in Taiwan [2]. According to a survey conducted in 2006, 89.5% of people with intellectual disabilities in Taiwan lived with family members and 78.0% had no paid employment [3]. People with intellectual disabilities had a shorter life expectancy than did the general population [4,5]. Standardized mortality ratios for adults with moderate or severe intellectual disabilities were 3 times higher than those for the general population [6].

A study in the Netherlands determined that people with intellectual disabilities had 2.5 times more health problems than did those without intellectual disabilities [7]. Numerous people with intellectual disabilities developed neurological, digestive, dermatological, and mental disorders, as well as obesity, diabetes, and cardiovascular disease [8-11]. People with intellectual disabilities might be unaware of physical problems and might have difficulty verbally expressing such conditions. Patients with intellectual disabilities are often rushed to hospitals for treatment when their physical conditions become severe. Therefore, people with intellectual disabilities must expend more time and effort in receiving medical care and may not be able to obtain required appropriate treatments [12-14].

Previous studies have demonstrated that the intellectually disabled persons were less likely to receive preventive health services than the others [15-18]. For instance, only 25% of women with learning disabilities in Exeter (a city in southwestern England) underwent cervical screening [19], and in Wales, only 31%–41% of people with learning disabilities received annual health assessments in 2006 and 2009 [20]. People with intellectual disabilities have substantial health needs, and have been reported to benefit from regular health assessments. A randomized controlled study conducted in Australia showed that people with intellectual disabilities who regularly received health assessments were newly diagnosed with diseases at a rate that was 1.6 times that of those who did not receive regular health assessments [21].

A study in the United States suggested that an increase in preventive services could avert the loss of more than 2 million life-years annually [22]. Increasing clinical preventive health services could effectively lower subsequent medical expenses [23-26]. Previous studies have indicated that sex, marital status [27], educational level, age, income, health status, severity of disability, and urbanization level influence the use of preventive health services among disabled people [28].

To reduce exorbitant medical expenses and improve unequal access to health care, free preventive health services for adults have been promoted since 1995 in Taiwan. The services include medical examinations, health education, blood, and urine tests. All adults aged over 40 years are accessible to this free service. Frequency limitations of this service varied according to different age ranges, i.e., once per three years for the persons aged 40–64 years and once per year for those who aged over 65 years. The examination outcomes are reported to patients, and primary care physicians suggest necessary additional diagnoses, treatments, or follow-ups. There were 21,042 adults with intellectual disabilities met the requirements in 2008. The purpose of this study was to explore of preventive health service utilization among these people and the factors associated with their use.

## Methods

### Data source and participants

According to the Disabled Welfare Law (1980), local governments in Taiwan provide support such as social welfare, special education, and health care to people with intellectual disabilities. Intelligence quotient (IQ) scores are diagnosed based on an official test administered by a psychologist and certified by the government; the scores are then confirmed by a doctor accredited by the government. If the IQ score of a person is below 70 (more than 2 standard deviations below the mean), the person is identified as having intellectual disabilities. Local governments certify disabled residents and report cases to the central government, and the Ministry of the Interior maintains a registry of certified cases. Intellectual disabilities are categorized according to four levels of severity, namely very severe (IQ: 5 standard deviations below the mean), severe (IQ: 4–5 standard deviations below the mean), moderate (IQ: 3–4 standard deviations below the mean), and mild (IQ: 2–3 standard deviations below the mean).

The study population was 21,042 people in Taiwan with intellectual disabilities, aged over 40 years, and registered with the Ministry of the Interior as of 2008 (Department of Statistics, 2008). Among them, 17.46%, 32.20%, 30.34%, and 20.00% were diagnosed with mild, moderate, severe, and very severe levels of intellectual disabilities, respectively.

The Health Promotion Administration (HPA) has provided free preventive health services for adults in Taiwan since 1995 and maintains a dataset of records of adults who have used such services. Since 1995, Taiwan has implemented the National Health Insurance (NHI) program; 99.68% of the residents are enrolled in the NHI program. The NHI program is a universal, comprehensive health insurance program with a considerably low copayment. The NHI Administration holds all medical claims data and publishes the National Health Insurance Research Dataset for academic research annually. In this study, three data sources were used: the 2006–2008 preventive health service dataset obtained from the HPA, medical claims data from the NHI Research Database provided by the Ministry of Health and Welfare, and information on disabled people from the 2008 Registry of Disabled People obtained from the Ministry of the Interior. The Statistics Center of the Department of Health, Taiwan, helped match the three datasets with personal identification numbers and provided a dataset that included that necessary information for this study. All personal identification information was deleted and personal privacy was protected. The institutional review board of China Medical University and Hospital approved this study (IRB No. CMU-REC-101-012).

### Description of variables

Variables in this study included demographics (e.g., gender, age, marital status, educational level, and aborigine status, economic conditions (e.g., premium-based monthly payroll, low-income household status), health status (with or without a catastrophic illness/injury), chronic diseases (including mental disorders, musculoskeletal system and connective tissue diseases, neurological disorders, cancers, blood and blood-forming organs diseases, circulatory system diseases, respiratory diseases, endocrine and metabolic diseases, digestive diseases, genitourinary system diseases, skin and subcutaneous tissue disease, diseases of eyes and auxiliary organs, ear and mastoid diseases, infectious diseases, and congenital malformations); environmental factors (i.e., urbanization of resident areas), severity of disability (i.e., very severe, severe, moderate, and mild), and utilization of adult preventive health services. Urbanization was categorized into eight levels. The first level was the area with the highest level of urbanization, whereas the eighth level was the region with the lowest level of urbanization. A low-income household was defined as a household in which the average monthly income per person was below the lowest living index, i.e., 60% of the living expenditure per person in the previous year in the local area of the household [29].

### Statistical analysis

A statistics software package (SAS 9.2) was used for data analysis. Descriptive statistics was used to describe the percentages of demographic characteristics, economic status, health status, environmental factors, levels of intellectual disability, and the utilization of preventive health services for adults. Chi-square test was used to determine the relationship between the utilization of preventive health services and these variables. Multivariate logistic regression analysis was applied to explore the factors associated with the use of adult preventive health services among the persons with intellectual disabilities. The full model approach was applied in logistic regression analysis. In this study, a *p* value of less than 0.05 was considered statistically significant.

## Results

### Basic characteristics of the participants

Among the 21,042 participants, males were the majority (52.97%, n =11,145) (Table 1). Those aged 45–49 years were predominant (26.66%, n =5,609). Most of the participants had a educational level of elementary school or lower (72.84%, n =15,327). Those with premium-based monthly payroll less than NT $15,840 (New Taiwan Dollars, NT$) (U.S. $1 = NT $30) accounted for the majority (51.69%, n =10,877). Regarding the relevant chronic diseases, intellectual disability with comorbid mental illness (26.44%) and circulatory system disease (23.93%) were ranked in the first and second places, respectively. Those with moderate level of severity of intellectual disabilities were predominant, accounting for 32.20% (n =6,775).

**Table 1:** Use of adult preventive health services among the intellectual disability: basic characteristics and bivariate analysis

			**Used**		**Did not use**		**χ**^**2**^
**Variables**	**N = 21042**	**%**	**n**_**1**_ **= 3503**	**%**	**n**_**2**_ **= 17539**	**%**	**p-value**
Overall rate of use				16.65			
Gender							< .001
Female	9897	47.03	1808	18.27	8089	81.73	
Male	11145	52.97	1695	15.21	9450	84.79	
Age							< .001
40-44 years	3753	17.84	628	16.73	3125	83.27	
45-49 years	5609	26.66	921	16.42	4688	83.58	
50-54 years	4420	21.01	774	17.51	3646	82.49	
55-59 years	3089	14.68	609	19.72	2480	80.28	
60-64 years	1817	8.64	367	20.20	1450	79.80	
65-69 years	1028	4.89	94	9.14	934	90.86	
≥ 70 years	1326	6.30	110	8.30	1216	91.70	
Educational level							0.535
Elementary school and under	15327	72.84	2566	16.74	12761	83.26	
Junior high school	2645	12.57	454	17.16	2191	82.84	
Senior (vocational) high school	550	2.61	80	14.55	470	85.45	
Junior college and university or above	99	0.47	16	16.16	83	83.84	
Unclear	2421	11.51	387	15.99	2034	84.01	
Marital status							< .001
Married	6386	30.35	1239	19.40	5147	80.60	
Unmarried	8463	40.22	1346	15.90	7117	84.10	
Divorced or widowed	677	3.22	139	20.53	538	79.47	
Unclear	5516	26.21	779	14.12	4737	85.88	
Level of urbanization^a^							< .001
Level one	1802	8.56	201	11.15	1601	88.85	
Level two	3394	16.13	553	16.29	2841	83.71	
Level three	2865	13.62	468	16.34	2397	83.66	
Level four	1809	8.60	295	16.31	1514	83.69	
Level five	3483	16.55	562	16.14	2921	83.86	
Level six	3006	14.29	589	19.59	2417	80.41	
Level seven	3173	15.08	569	17.93	2604	82.07	
Level eight	1510	7.18	266	17.62	1244	82.38	
Premium-based monthly payroll							< .001
Dependents	5303	25.20	720	13.58	4583	86.42	
< 15,840	10877	51.69	1898	17.45	8979	82.55	
16,500-22,800	4143	19.69	770	18.59	3373	81.41	
24,000-28,800	358	1.70	57	15.92	301	84.08	
30,300-36,300	210	1.00	31	14.76	179	85.24	
> 38,200	151	0.72	27	17.88	124	82.12	
Low-income household							< .001
Yes	3713	17.65	799	21.52	2914	78.48	
No	17329	82.35	2704	15.60	14625	84.40	
Aborigine							0.001
Yes	267	1.27	66	24.72	201	75.28	
No	20775	98.73	3437	16.54	17338	83.46	
Catastrophic illness/injury							< .001
Yes	2538	12.06	602	23.72	1936	76.28	
No	18504	87.94	2901	15.68	15603	84.32	
Relevant chronic diseases							
Cancer							0.344
Yes	266	1.26	50	18.80	216	81.20	
No	20776	98.74	3453	16.62	17323	83.38	
Endocrine and metabolic disease							< .001
Yes	4319	20.53	1324	30.66	2995	69.34	
No	16723	79.47	2179	13.03	14544	86.97	
Mental illness							< .001
Yes	5564	26.44	1416	25.45	4148	74.55	
No	15478	73.56	2087	13.48	13391	86.52	
Disease of the nervous system							< .001
Yes	2636	12.53	686	26.02	1950	73.98	
No	18406	87.47	2817	15.30	15589	84.70	
Disease of the circulatory system							< .001
Yes	5035	23.93	1435	28.50	3600	71.50	
No	16007	76.07	2068	12.92	13939	87.08	
Disease of the respiratory system							< .001
Yes	3047	14.48	886	29.08	2161	70.92	
No	17995	85.52	2617	14.54	15378	85.46	
Disease of the digestive system							< .001
Yes	4861	23.10	1398	28.76	3463	71.24	
No	16181	76.90	2105	13.01	14076	86.99	
Disease of the urinary system							< .001
Yes	459	2.18	136	29.63	323	70.37	
No	20583	97.82	3367	16.36	17216	83.64	
Disease of the skeletal and muscular system and connective tissue							< .001
Yes	3830	18.20	1078	28.15	2752	71.85	
No	17212	81.80	2425	14.09	14787	85.91	
Disease of the eyes and auxiliary organs							< .001
Yes	640	3.04	161	25.16	479	74.84	
No	20402	96.96	3342	16.38	17060	83.62	
Infectious diseases							< .001
Yes	839	3.99	210	25.03	629	74.97	
No	20203	96.01	3293	16.30	16910	83.70	
Congenital malformation							< .001
Yes	277	1.32	71	25.63	206	74.37	
No	20765	98.68	3432	16.53	17333	83.47	
Skin and subcutaneous tissue disorders							< .001
Yes	1861	8.84	551	29.61	1310	70.39	
No	19181	91.16	2952	15.39	16229	84.61	
Diseases of the blood and blood-forming organs							< .001
Yes	761	3.62	227	29.83	534	70.17	
No	20281	96.38	3276	16.15	17005	83.85	
Diseases of the ear and mastoid process							< .001
Yes	735	3.49	232	31.56	503	68.44	
No	20307	96.51	3271	16.11	17036	83.89	
Severity of intellectual disability							< .001
Mild	3673	17.46	712	19.38	2961	80.62	
Moderate	6775	32.20	1194	17.62	5581	82.38	
Severe	6385	30.34	1015	15.90	5370	84.10	
Very severe	4209	20.00	582	13.83	3627	86.17	

^a^Level one: the most urbanized areas.

### The utilization of adult preventive health services among the participants

As presented in Table 1, 16.65% (n =3,503) of participants aged over 40 years used the adult preventive health services. Of them, more females (18.27%) used the services than males (15.21%, *p* < 0.001). Those aged 60–64 years had the highest utilization (20.20%, *p* < 0.001). According to the levels of urbanization, those living in the areas of 6^th^ level of urbanization had the highest utilization (19.59%) whereas those living in urbanization of first level (most urban) had the lowest utilization (11.15%) (*p* < 0.001). The participants with any catastrophic illness/injury used more preventive health services than those without (23.72% vs. 15.68%, *p* < 0.001). Among those with relevant comorbid diseases, those having the highest utilization rate were those with diseases of the ear and mastoid process (31.56%), followed by those with endocrine and metabolic disease (30.66%), and those with cancers had the lowest utilization rate (18.80%) compared to the others. The persons with more severe level of intellectual disability were the lower frequent users of services (*p* < 0.001), indicating that the lowest users were those with very severe level (13.83%) and those with highest utilization rate were the subgroup of mild level of disabilities (19.38%).

### Factors influencing the utilization of adult preventive health services among participants

The results of analyzing variables associated with the utilization of adult preventive health services are shown in Table 2. The factors significantly influencing the utilization included gender, age, marital status, urbanization of resident area, premium-based monthly payroll, low-income household status, catastrophic illness/injury status, relevant chronic diseases, and severity of intellectual disabilities. After controlling for other variables, males were 0.87 times less likely to use adult preventive health services than females (OR = 0.87, 95% CI = 0.80-0.95). When those aged 40–44 years were used as a reference group, the groups aged 65–69 years or ≥ 70 years had significantly lower probabilities to use the services (OR = 0.35, 95% CI = 0.28-0.44; OR = 0.33, 95% CI = 0.26-0.42). Furthermore, the probability of using the services increased with decreasing urbanization of resident areas. Those living in areas with the 6^th^ level of urbanization were 2.47 times more likely to use the services than those living in the area of first level (most urban) (OR = 2.47, 95% CI = 2.06-2.97).

**Table 2:** Factors influencing the intellectual disability to use adult preventive health services: logistic regression analysis

	**Unadjusted**				**Adjusted**			
**Variable name**	**OR**	**95% CI**		**p-value**	**OR**	**95% CI**		**p-value**
Gender								
Female	-	-	-	-	-	-	-	-
Male	0.80	0.75	0.86	< .001*	0.87	0.80	0.95	0.001*
Age								
40-44 years	-	-	-	-	-	-	-	-
45-49 years	0.98	0.88	1.09	0.689	0.92	0.82	1.03	0.140
50-54years	1.06	0.94	1.19	0.353	0.94	0.83	1.06	0.290
55-59 years	1.22	1.08	1.38	0.001*	1.00	0.88	1.15	0.950
60-64 years	1.26	1.09	1.45	0.002*	1.03	0.89	1.21	0.677
65-69 years	0.50	0.40	0.63	< .001*	0.33	0.26	0.42	< .001*
≥ 70 years	0.45	0.36	0.56	< .001*	0.35	0.28	0.44	< .001*
Educational level								
Elementary school and under	-	-	-	-	-	-	-	-
Junior high school	1.03	0.92	1.15	0.591	1.03	0.91	1.16	0.676
Senior (vocational) high school	0.85	0.67	1.08	0.175	0.78	0.60	1.01	0.057
Junior college and university or above	0.96	0.56	1.64	0.878	1.11	0.63	1.96	0.714
Unclear	0.95	0.84	1.06	0.353	0.98	0.87	1.12	0.790
Marital status								
Married	-	-	-	-	-	-	-	-
Unmarried	1.27	1.17	1.39	< .001*	1.07	0.97	1.19	0.168
Divorced or widowed	1.37	1.12	1.66	0.002*	1.16	0.94	1.43	0.179
Unclear	0.87	0.79	0.96	0.004*	0.79	0.71	0.88	< .001*
Level of urbanization^a^								
Level one	-	-	-	-	-	-	-	-
Level two	1.55	1.31	1.84	< .001*	1.79	1.49	2.15	< .001*
Level three	1.56	1.30	1.86	< .001*	1.85	1.53	2.23	< .001*
Level four	1.55	1.28	1.88	< .001*	1.90	1.55	2.33	< .001*
Level five	1.53	1.29	1.82	< .001*	1.91	1.59	2.29	< .001*
Level six	1.94	1.63	2.31	< .001*	2.47	2.06	2.97	< .001*
Level seven	1.74	1.47	2.07	< .001*	2.19	1.82	2.64	< .001*
Level eight	1.70	1.40	2.08	< .001*	2.08	1.68	2.57	< .001*
Premium-based monthly payroll								
< 15,840	-	-	-	-	-	-	-	-
Dependent population	0.74	0.68	0.82	< .001*	0.90	0.81	1.01	0.061
16,500-22,800	1.08	0.98	1.19	0.104	1.16	1.04	1.29	0.011*
24,000-28,800	0.90	0.67	1.19	0.453	1.03	0.76	1.40	0.836
30,300-36,300	0.82	0.56	1.20	0.310	0.91	0.61	1.37	0.653
> 38,200	1.03	0.68	1.57	0.890	1.12	0.72	1.74	0.631
Low-income household								
No	-	-	-	-	-	-	-	-
Yes	1.48	1.36	1.62	< .001*	1.27	1.14	1.42	< .001*
Aborigine								
No	-	-	-	-	-	-	-	-
Yes	1.66	1.25	2.19	0.000*	1.29	0.95	1.75	0.103
Catastrophic illness/injury								
No	-	-	-	-	-	-	-	-
Yes	1.67	1.51	1.85	< .001*	1.22	1.08	1.38	0.002*
Relevant chronic diseases								
Cancer	1.16	0.85	1.58	0.344	0.77	0.55	1.08	0.135
Endocrine and metabolic disease	2.95	2.73	3.19	< .001*	1.79	1.63	1.96	< .001*
Mental illness	2.19	2.03	2.36	< .001*	1.33	1.21	1.46	< .001*
Disease of the nervous system	1.95	1.77	2.14	< .001*	1.11	0.99	1.24	0.067
Disease of the circulatory system	2.69	2.49	2.90	< .001*	1.57	1.43	1.72	< .001*
Disease of the respiratory system	2.41	2.21	2.63	< .001*	1.38	1.24	1.53	< .001*
Disease of the digestive system	2.70	2.50	2.92	< .001*	1.49	1.36	1.63	< .001*
Disease of the urinary system	2.15	1.76	2.64	< .001*	0.97	0.77	1.21	0.766
Disease of the skeletal and muscular system and connective tissue	2.39	2.20	2.59	< .001*	1.31	1.19	1.45	< .001*
Disease of the eyes and auxiliary organs	1.72	1.43	2.06	< .001*	0.94	0.77	1.15	0.549
Infectious diseases	1.71	1.46	2.01	< .001*	0.98	0.82	1.16	0.776
Congenital malformation	1.74	1.33	2.29	< .001*	1.05	0.78	1.41	0.748
Skin and subcutaneous tissue disorders	2.31	2.08	2.57	< .001*	1.29	1.14	1.46	< .001*
Diseases of the blood and blood-forming organs	2.21	1.88	2.59	< .001*	1.18	0.99	1.40	0.065
Diseases of the ear and mastoid process	2.40	2.05	2.82	< .001*	1.08	0.91	1.29	0.382
Severity of intellectual disability								
Mild	-	-	-	-	-	-	-	-
Moderate	0.89	0.80	0.99	0.026*	0.88	0.79	0.98	0.020*
Severe	0.79	0.71	0.87	< .001*	0.82	0.73	0.92	0.001*
Very severe	0.67	0.59	0.75	< .001*	0.75	0.66	0.86	< .001*

^a^Level one: the most urbanized areas.

*p < 0.05.

Using those with less than NT$ 15,840 of premium-based monthly payroll as a reference group, the group with NT$ 16,500-22,800 was more likely to use the adult preventive health services (OR = 1.16, 95% CI = 1.04-1.29). Those with low-income household status were more likely to use services than those without low-income household status (OR =1.27, 95% CI = 1.14-1.42). Those with catastrophic illness/injury were 1.22 times (95% CI =1.08-1.38) more likely to use the services than those without. In addition, those having endocrine and metabolic diseases (OR = 1.79, 95% CI = 1.63-1.96) or circulatory system diseases (OR = 1.57, 95% CI = 1.43-1.72) were more likely to use the services than those who had not. The probability of using the services decreased with increasing severity of intellectual disabilities. Those with very severe intellectual disability were 0.75 times less likely to use the services than those with mild level of severity (95% CI = 0.66-0.86).

## Discussion

In 2004, the overall rate of use of adult preventive health services among those aged 40 to 64 years was 42%, while the rate of use among those aged 65 years or older was 38% [27]. However, only 16.65% of people with intellectual disabilities aged over 40 years used the preventive health services in the years 2006–2008, which was much lower than that of the general population. Another study indicated that, in Taiwan, among the disabled people using adult preventive health services, people suffering from chronic epilepsy had the highest use rate (23.33%), whereas disabled people with major organ malfunction had the lowest use rate (10.21%) [27].

The results of this study indicated that the utilization of adult preventive health services were not significantly associated with aborigine status and educational level. The finding of females with higher probability to use the services than males was consistent with previous studies reporting the utilization of relevant preventive health services [30-32]. Those aged ≥ 65 years were less likely to use the services than the others, which might be associated with many elderly persons with intellectual disabilities living in psychiatric hospitals, nursing facilities, and nursing homes [33].

People with an “unclear” marital status exhibited the lowest use rate. Most people with this status may have been unmarried, and unmarried people exhibited the lowest use rate compared with other marital status groups. The probability of using preventive health services increased with decreasing urbanization of resident areas. Generally, people living in urban areas have more convenient transportations and can get faster access to medical resources than those living in rural areas. However, the Bureau of National Health Insurance (NHI) initiated an Integrated Delivery System program in 1999 that covered all 48 mountainous and island districts. Under the program, NHI-contracted hospitals are responsible for providing medical care, including outpatient care, emergency services, and specialty services. Health care, which consists of outpatient care, preventive care, disease screening, and health education, is provided from mobile vehicles. The mobile care services were provided in mountain areas and offshore islands to narrow the health care disparities. For those with intellectual disabilities living in rural areas, it was common that they and their families went together to receive preventive health services when the mobile care services were provided. As the result, a higher probability of using the services was observed for the persons with intellectual disabilities living in rural areas than those in urban areas. The probability of using preventive health services was found lower for the participants living in the most urbanized areas in this study. It was more likely due to better medical resources and higher accessibility in urban areas. They could obtain necessary medical treatments once they were ill. The high accessibility resulted in less attention being paid to use preventive health services by the participants living in urbanized areas.

Our results indicated that those with a low-income household status were more likely to use the services than the others, which was inconsistent with previous studies reporting that higher incomes were associated with more frequent use of preventive health services [34-36]. This finding may be resulted from the improvement in eliminating health inequalities between rich and poor populations, as a consequence of implementation of the NHI in Taiwan since 1995.

Aboriginal persons with intellectual disabilities had similar probability of using the services compared to non-aborigines. The results reflected the outcomes of efforts to improve health for vulnerable populations, and to eliminate gaps of health care for the minority in Taiwan. The probability of using the services was higher in those with any catastrophic illness/injury than those without. It was possible that their families were more concerned with changes in their health status for those participants with a catastrophic disease. Therefore, they paid more attentions and used more preventive health services. In consistent with previous studies [37], we also found that those with very severe level of intellectual disability used less preventive health services. The cognitive and language skills for the persons with very severe intellectual disabilities are lower compared with those with moderate and mild levels of severity. They need assistance and company of other people, which lead to more difficulties in using preventive health services.

This study was limited by the sources of data, which did not include information pertaining to personal health beliefs or health behaviors. The lack of objective information on household income in our dataset is regarded as a limitation of this study.

Although health policy has established strategies for eliminating health inequalities that affect people with intellectual disabilities in Taiwan, preventive health services must be markedly improved for people with intellectual disabilities. According to the findings, conducting publicity through the media and educating the public on using adult preventive health services are suggested. In addition, health care organizations should aggressively encourage and arrange free preventive health services for people with intellectual disabilities when they visit physicians, particularly for groups that were determined not to use such services.

## Conclusions

This study demonstrated that the significant factors influencing the utilization of adult preventive health services for the persons with intellectual disabilities included gender, age, and urbanization of resident areas, premium-based payroll, low-income household status, marital status, catastrophic illness/injury status, relevant chronic diseases, and severity of intellectual disabilities. Non-significant factors were aboriginal status and educational level. Those with lower use of preventive health services were characterized by male gender, aged ≥ 65 years, high school education, unmarried, living in urban areas, with skin and blood-forming organs diseases, and with very severe intellectual disabilities.

## Abbreviations

CI: Confidence interval; HPA: Health Promotion Administration; IQ: Intelligence quotient; NHI: National Health Insurance; NT$: New Taiwan Dollar; OR: Odds Ratio; SAS: Statistics Analysis System.

## Competing interests

The authors declare that they have no competing interests.

## Authors’ contributions

WCT and PTK conducted the study design. SMY and LTC drafted the manuscript. PTK and SMY conducted the statistical analysis. WCT was the supervisor of the study and revised the manuscript critically for important intellectual content. All authors read and approved the manuscript.

## Pre-publication history

The pre-publication history for this paper can be accessed here:

http://www.biomedcentral.com/1472-6963/14/248/prepub
